# Relative survival in patients with dementia with Lewy bodies and Parkinson’s disease dementia

**DOI:** 10.1371/journal.pone.0202044

**Published:** 2018-08-10

**Authors:** Victoria Larsson, Gustav Torisson, Elisabet Londos

**Affiliations:** 1 Clinical Memory Research Unit, Department of Clinical Sciences Malmo, Skåne University Hospital, Malmö, Sweden; 2 Department of Infectious Diseases, Skåne University Hospital, Malmö, Sweden; Oslo Universitetssykehus, NORWAY

## Abstract

**Introduction:**

The understanding of survival in dementia with Lewy bodies (DLB) and Parkinson’s disease dementia (PDD) is limited, as well as the impact of these diagnoses in an ageing co-morbid population.

**Methods:**

A retrospective study of 177 patients who received a DLB or PDD diagnosis between 1997–2014 at the Memory Clinic in Malmö, Sweden. Relative survival was evaluated by adjusting all-cause survival for expected survival, estimated from population life-tables, matched by sex, age and calendar year. Predictors of relative survival were investigated using multivariate regression modelling.

**Results:**

At follow-up, 143 (81%) patients were deceased with a median survival of 4.1 years (IQR 2.6–6.0). After 10-years follow-up, the standardized mortality ratio was 3.44 (95% CI 2.92–4.04). Relative survival was worse with younger age at diagnosis (excess hazard ratio [eHR] 0.91, 95% CI 0.88–0.94 per year of age), female sex (eHR 1.45, 95% CI 1.01–2.09) and lower mini-mental state examination (eHR 0.93, 95% CI 0.90–0.96). Subgroup analysis (n = 141) showed higher mortality in DLB patients who were positive for *APOE* ɛ4 (eHR 2.00, 95% CI 1.35–2.97).

**Conclusion:**

The mortality is over three-times higher in patients diagnosed with dementia with Lewy bodies and Parkinson’s disease dementia during a ten-year follow-up, compared to persons in the general population. Excess mortality is found primarily in younger patients, females and carriers of *APOE* ε4. Further research is needed regarding survival and possible interventions, including disease-modifying treatments, to improve care for this patient group.

## Introduction

Dementia with Lewy bodies (DLB) is a common neurodegenerative disorder associated with neuronal alpha-synuclein inclusions, and a complex clinical manifestation consisting of cognitive decline, parkinsonism, fluctuations, visual hallucinations, REM sleep behavior disorder and autonomic dysfunction [1]. These clinical and pathological hallmarks are also found in the large group of patients with Parkinson’s disease who develop dementia (PDD), with the only prominent difference being the earlier debut of motor disturbances [2].

A diagnosis of dementia is associated with increased mortality, but survival estimates in previous research have varied greatly with dementia type, sex and study design [3]. In the general dementia population increased age at onset, male sex, multiple comorbidities and functional impairment have been linked to shorter survival [4]. However, the understanding of survival in DLB and PDD is limited and studies are lacking [5]. Survival has been ranging between 1.9 to 7.7 years from diagnosis in DLB patients [4, 6–14], with some studies showing worse survival than in Alzheimer’s disease (AD) [11, 15] and in Parkinson’s disease (PD) [11, 12]. Risk factors that have been specifically attributed to a higher mortality in DLB include co-morbid Alzheimer pathology, indicated by presence of *APOE* ε4 allele [8, 16], decreased hippocampal volume [17] and CSF AD profile [18, 19].

To capture the true impact of the disease of interest on mortality, ideally only the cause-specific mortality should be measured. The main obstacle of cause-specific survival is poor reporting of cause of death. In patients with dementia the diagnosis is frequently missed on the death certificate [20], particularly in those with Lewy body dementia [21]. Therefore, the majority of survival studies report on the all-cause mortality. However, this does not separate deaths occurring due to the disease of interest and deaths unrelated to the disease of interest. In an aged and comorbid population, other causes of death would be expected.

An alternative measure is relative survival, or excess mortality. This estimates disease-specific mortality by adjusting the all-cause mortality with the expected mortality in the general population [22]. Life-tables from the general population are used to estimate the expected mortality based on age, sex and calendar year. Although most commonly applied in population-based cancer studies, other fields have also started to use these methods [23–25]. One advantage is that excess mortality associated with the diagnosis is measured irrespective if this is directly or indirectly attributable to the condition. For example, in DLB and PDD patients there is an increased risk of falls and pneumonia, which might be fatal and therefore indirectly attributable to the diagnosis.

Our aim was to investigate survival in a group of DLB and PDD patients compared to the general population, and to identify factors contributing to excess mortality by using relative survival methods.

## Materials and methods

### Study setting, design and participants

We retrospectively identified all patients who received a diagnosis of DLB or PDD at the Memory Clinic in Malmö, Skåne University Hospital, Sweden, between 1997–2014. All data was collected from the clinic’s electronical medical records.

The Memory Clinic in Malmö is a secondary care clinic specializing in cognitive disorders, situated in Sweden’s third largest city Malmö (population size was 301,706 in 2015) [26]. According to regional guidelines, all patients with suspected DLB or PDD should be referred to the Memory Clinic from their primary care physician or other care institutions, where the patients will receive a diagnosis after a structured medical history, physical, psychiatric and neurological examination, cognitive testing, blood samples, CT or MRI of the brain. Further investigations, such as EEG, molecular imaging or CSF analysis, are conducted when judged appropriate by the responsible clinician. A small number of patients are also referred for post-mortem examination.

Patients with a diagnostic code of DLB or PDD (ICD-10 codes F02.8 G31.8A or F02.3 G20.9) in the electronic medical records were identified up until 31st of December 2014. Author VL reviewed the medical records to confirm the diagnoses according to consensus criteria for DLB [27] and criteria for PDD [28]. Patients lacking information to fulfill the diagnostic criteria or with a diagnosis prior to the introduction of electronic medical records in 1997 were excluded.

Data was collected regarding demographics, time of first visit, time of diagnosis, apolipoprotein genotype and the mini-mental state examination (MMSE) score at the time of diagnosis [29]. To obtain a measure of combined comorbidity, Charlson comorbidity index (CCI) was calculated according to the original protocol [30], using the comorbidities listed in the electronic medical records at the time of diagnosis. The CCI includes 19 different conditions with varying weights, whereby dementia is included and has a weight of 1. Results from ^[123I]^FP-CIT SPECT (DaTscan) and autopsy were also collected when available.

### Statistical analysis

The goal of this study was to investigate survival in DLB and PDD patients using overall and relative survival methods. Statistical methods are explained in detail in S1 File. Modelling was carried out in R and the script can be found in S2 File [31].

All variables in statistical models were analyzed initially in their original form on their original scale, except CCI which was dichotomized. Year of diagnosis was included as a variable to adjust for survival effects that could be dependent on improvements in care over time.

Survival status was determined from the Swedish Population Registry, and survival time was defined as the time from diagnosis to death or until last follow-up (17 May 2017). Cox proportional hazards modelling was used to determine the effect of covariates on overall survival using both bivariate and multivariate models. The assumption of proportional hazards was tested using Schoenfeld residuals.

The impact of the diagnosis was estimated using the standardized mortality ratio (SMR) [32]. This gives an estimate of the likelihood of death in a certain time period in patients with the diagnosis of interest compared to the general population.

Relative survival modelling was used to separate mortality due to the disease of interest, from mortality due to all other causes. The cumulative relative survival function is defined as the ratio between observed survival and expected survival in the background population [33]. Similarly, excess mortality in the cohort of interest is the difference between total observed mortality in the cohort and the expected mortality [22].

Expected survival was calculated with the recommended Hakulinen method [34, 35], using life-tables from the Swedish population, obtained from and the Human Mortality Database (www.mortality.org) and split by sex, age and calendar year. Relative survival curves were calculated using the Pohar-Perme method and relative regression modelling was performed using transformed survival times [36]. This yields excess hazard ratios (eHR), similar to standard hazard ratios in Cox survival models, allowing estimation of covariate effect on excess hazard, e.g. the effect of sex on excess mortality. An eHR of > 1 indicates excess mortality compared to the general population, whilst < 1 signifies reduced mortality. As with Cox regression analysis, relative survival allows for multivariate modelling and adjusting for several cofactors. The proportional hazards assumption for relative survival models was tested forming a Brownian Bridge (see Fig D in S1 File) [37]. Separate analysis was performed for the subgroup with *APOE* genotyping, including interaction between *APOE* ɛ4 and diagnosis.

### Ethical considerations

In this retrospective study, only regular clinical data was reviewed. All patients were included in longitudinal follow-up programs and most had previously consented to prospective studies using the very same clinical data. At the time of conducting the study, 80% were deceased, and thus not able to give further consent. In view of this, the local institutional review board (IRB) did not consider further consent to be applicable for this retrospective study, but instead recommended an opt-out strategy consisting of an advertisement in a local newspaper. The Regional Ethics Committee in Lund, Sweden, approved this study.

## Results

### Baseline demographics

**Fig 1:**
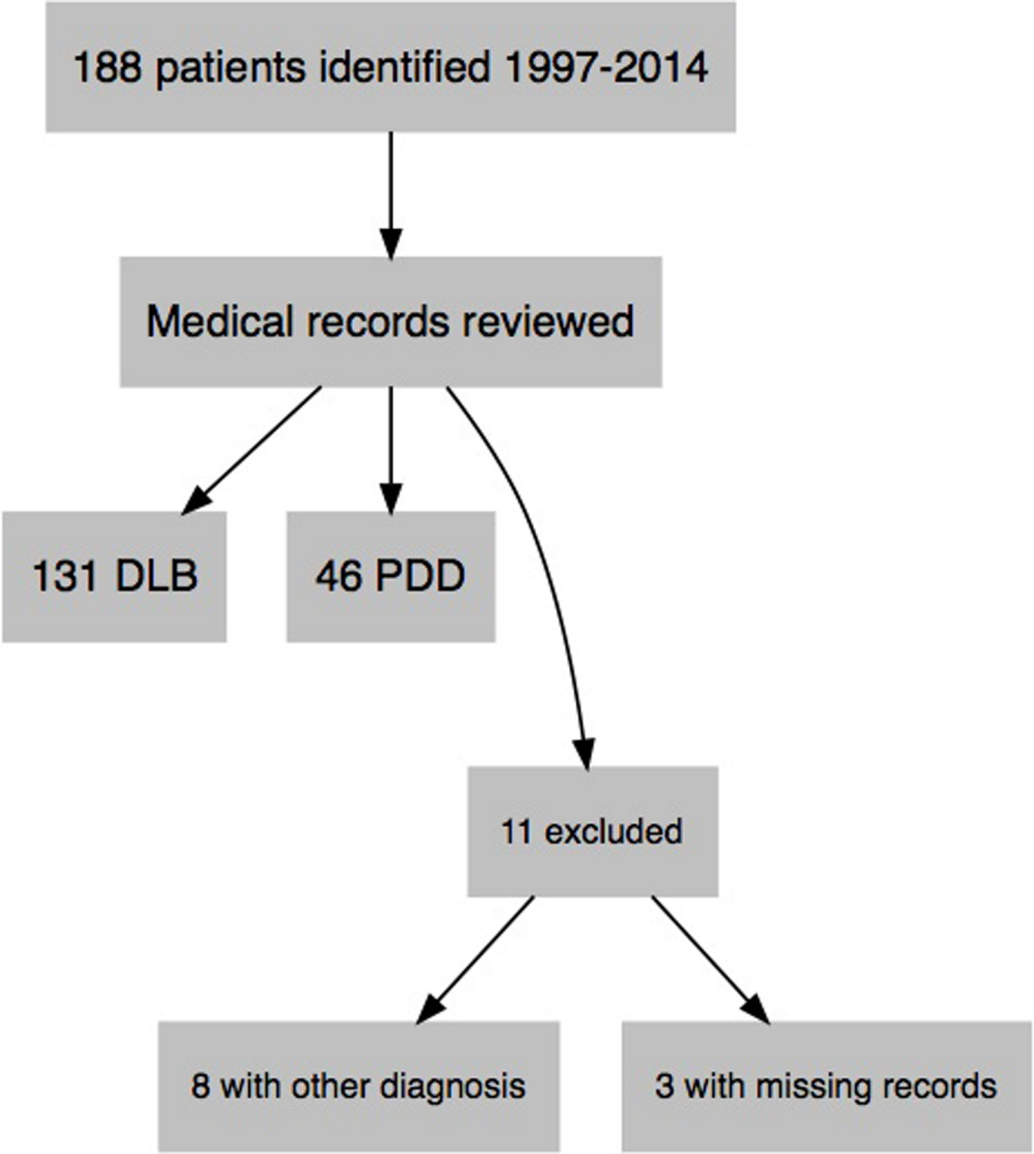
Patient flowchart.

188 patients were identified with a diagnosis of DLB or PDD in the electronic medical journal system. The medical journals were reviewed to verify the diagnoses according to consensus criteria, identifying in total 131 DLB patients and 46 PDD patients. Eight patients had diagnoses other than DLB and PDD; Parkinson’s disease without dementia (2), Alzheimer’s disease with vascular disease (2), progressive supranuclear palsy (2), dementia pugilistica (1), normal pressure hydrocephalus (1). Three patients had no available electronic medical records from the time of diagnosis and were therefore excluded (Fig 1).

Baseline demographics are described in Table 1. All patients had a minimum weighted comorbidity index of 1 because of dementia diagnosis, and 66.7% of patients had no other significant comorbidities. ^[123I]^FP-CIT SPECT scan was positive in 80/85 patients. Post-mortem diagnosis was available for 13 DLB patients, confirming the diagnosis in all cases.

**Table 1:** Baseline demographics.

	All patients (n = 177)
**Age at diagnosis, years (±SD)**	75.7 (5.8)
**Time from first assessment to diagnosis, months (IQR)**	11 (0–31)
**Year at diagnosis (SD)**	2007 (4)
**Male sex (%)**	114 (64.4)
**Diagnosis DLB:PDD (%)**	131:46 (74:26)
**Residency (%)**	
**With another adult**	122 (68.9)
**Alone**	41 (23.2)
**Nursing home**	14 (7.9)
**CCI (%)**	
**1 point**	118 (66.7)
**2 points**	39 (22.0)
**3 points**	14 (7.9)
**4 points**	5 (2.8)
**5 points**	0 (0)
**6 points**	1 (0.6)
***APOE* ɛ4 carrier (%), n = 141**	67 (47.5)
**MMSE at diagnosis, points (±SD)**	22.1 (4.9)

DLB, dementia with Lewy bodies; PDD, Parkinson’s disease dementia; CCI, Charlson co-morbidity index; *APOE*, apolipoprotein E; MMSE, mini-mental state examination.

### Survival analysis

A total of 143 patients (80.7%) were deceased at follow-up. The median survival was 4.1 years (IQR 2.6–6.0) for the overall group; 4.1 (IQR 2.2–5.3) in males and 4.0 (IQR 2.6–5.6) in females. The 5-year and 10-year SMR was 3.02 (95% CI 2.46–3.67) and 3.44 (95% CI 2.92–4.04) respectively for DLB/PDD patients.

**Fig 2:**
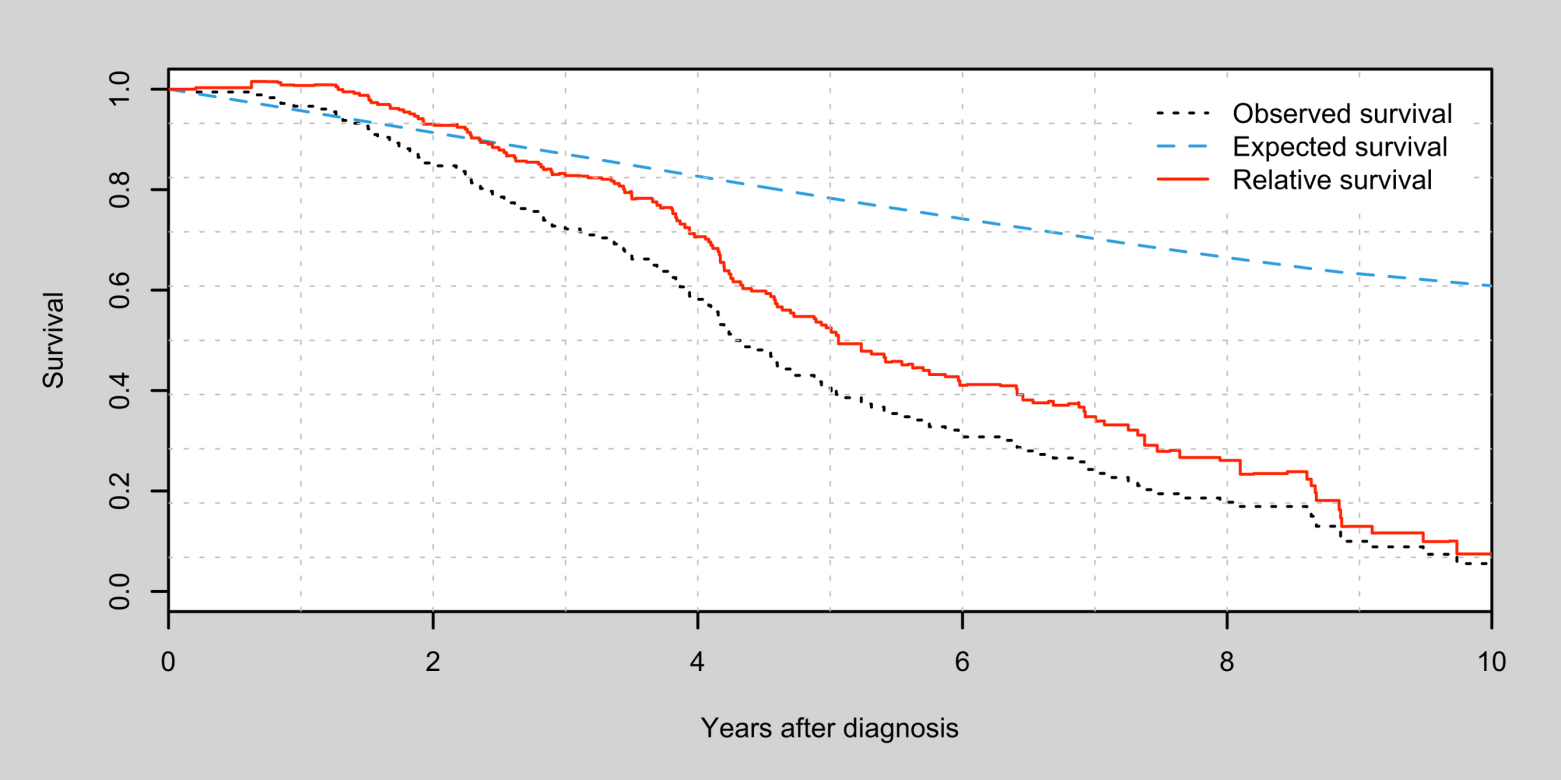
Survival curves in all patients. Comparison of observed, expected and relative survival times after diagnosis.

The observed, expected and relative survival rates for the patient group is illustrated in Fig 2. Compared to SMR, these measures provide information of both survival time and background mortality [38]. The observed 5-year and 10-year survival was 40.5% and 5.6% respectively, compared to the expected survival rates of 78% at 5 years and 62% at 10 years. Adjusting for expected mortality results in a 5-year and 10-year relative survival rate of 52.5% and 9.1% respectively (see Table C in S1 File for cumulative survival rates).

### Regression models

Unadjusted Cox regression showed that older age and lower MMSE predicted worse overall survival (Table A in S1 File). Performing a multivariable Cox regression model and adjusting for the baseline variables did not influence the results (Table B in S1 File).

Excess mortality compared to the general population is demonstrated in Table 2, showing a multivariable model adjusting for all baseline variables (except *APOE* ɛ4, see instead subgroup analysis below). In comparison to Cox regression, age is negatively associated with excess mortality (eHR 0.91), which can be attributed to higher expected survival in younger patients. Conversely, excess mortality was significantly increased in females (eHR 1.45), attributable to the increased expected survival in this group.

**Table 2:** Multivariable relative survival model. Predictors of relative survival expressed by excess hazard ratios (eHR) for all baseline variables.

Variable	*β*	eHR	95% CI		SE	z	p value
**Age at diagnosis, years**	-0.09	0.91	0.88	0.94	0.02	-5.34	<0.0001
**Year at diagnosis**	0.02	1.02	0.97	1.08	0.03	0.84	0.40
**Presentation to diagnosis, months**	0.00	1.00	0.99	1.02	0.01	0.50	0.62
**Sex** **0 = male, 1 = female**	0.37	1.45	1.01	2.09	0.19	1.99	<0.05
**Diagnosis** **0 = DLB, 1 = PDD**	-0.08	0.92	0.62	1.37	0.20	-0.42	0.68
**Nursing home residency** **0 = no, 1 = yes**	0.35	1.42	0.77	2.65	0.32	1.12	0.26
**CCI** **0 = 0–1, 1 = 2/more**	-0.04	0.96	0.67	1.39	0.19	-0.21	0.84
**MMSE score at diagnosis**	-0.07	0.93	0.90	0.96	0.02	-4.35	<0.0001

*β*, regression coefficient; S.E, standard error; eHR, excess hazard ratio; CI, confidence interval, MMSE, mini-mental state examination.

### Comparison of survival in DLB and PDD

No significant differences in survival was seen between DLB and PDD patients. Median survival time in DLB and PDD was comparable at 4.2 and 4.0 years respectively, and the 10-year SMR was 3.36 (95% CI 2.75–4.06) for DLB patients and 3.95 (95% CI 2.94–5.20) for PDD patients. We have included the observed (Kaplan-Meier) and relative survival curves comparing DLB and PDD in Fig C in S1 File, showing no significant differences for using either method. Similarly, no differences can be found when applying Cox regression or relative regression analyses (Table A and D in S1 File). This was true also after adjusting survival for sex and age and diagnosis (Table F in S1 File).

### Subgroup analysis with APOE ɛ4

In those with *APOE* genotyping, 47.5% had one or two *APOE ɛ4* alleles. Patients with missing analysis for *APOE* were older and more commonly PDD patients (see Table G in S1 File). Comparison of baseline variables between carriers and non-carriers of *APOE* ɛ4 showed no significant differences (Table H in S1 File).

Unadjusted bivariate Cox regression showed an HR of 1.45 (95% CI 1.03–2.16) in carriers of *APOE* ɛ4. Investigating the interaction between diagnosis and *APOE* ɛ4, displayed that patients with DLB and *APOE* ɛ4 had increased hazard (HR 1.85, 95% CI 1.25–2.74) in an age- and sex-adjusted model. A significant increased hazard was not found in *APOE* ɛ4 PDD patients (HR 1.4, 95% CI 0.73–2.65).

In the relative survival model, adjusting for expected mortality, eHR was 1.77 (95% CI 1.22–2.57) for *APOE* ɛ4 carriers. Testing the model with the interaction between DLB and *APOE* ɛ4 showed an eHR of 2.00 (95% CI 1.35–2.97). No significant effect was seen for PDD patients (eHR 1.04, 95% 0.49–2.24).

## Discussion

In this study, we have investigated the survival in DLB/PDD, showing that these patients are over three times more likely to die than an age-and sex-matched population during the same time period. By using relative regression models, we have also demonstrated how patients who are females, are younger and have suspected concomitant AD pathology are at risk of additional excess mortality compared to the general population.

To assess the true impact of a diagnosis on survival, it is relevant to assess the relationship between observed and expected survival. If the mortality seen in DLB/PDD patients would be due to ageing alone, then the observed and expected mortality would be similar. We can see in Fig 2 that this is not the case; the observed survival in DLB/PDD patients is significantly worse than what would be expected from the background population. This indicates that DLB/PDD are diagnoses significantly shortening the lives of those affected. This can also be concluded from an SMR of 3.44, which is similar to the impact of long-term mortality in ischemic stroke [39].

By using relative survival methods, we have also been able to show that a diagnosis of DLB/PDD has a larger impact on female life expectancy than male (excess mortality of 1.45). Male sex has been considered a key risk factor for earlier mortality in dementia, whilst evidence specifically for DLB is ambiguous [8, 40]. A recently large study in DLB indicate worse survival in males, although not statistically significant in an adjusted model [15]. However, these studies have only measured observed survival, and thus not accounted for the longer life expectancy typically seen in females compared to men. Therefore, although the numerical survival time was nearly identical between males and females after diagnosis, excess mortality is found to be increased in females. Similarly, despite having a longer survival time, a younger age at diagnosis is associated with excess mortality since a life expectancy is reduced to a greater extent.

In our patient population, 47.5% were *APOE* ε4 carriers, which is similar to rates found in both pathologically verified and clinical cohorts [16–18, 41]. Patients with DLB who were *APOE* ε4 carriers had a worsened survival compared to non-carriers, which support work by others [8, 16]. The worsened survival might be due to increasing likelihood of amyloid-*β* deposition and consequently Alzheimer-pathology, leading to an increased total pathological burden, suggested in other studies [17–19, 40, 42]. Similar to other research findings [42], we did not see an effect of *APOE* ε4 in PDD patients, though this needs to be interpreted with caution, considering the small number of PDD patients in our analysis.

Only a few studies with varying study designs have compared survival in DLB/PDD to the general population, which complicates comparison. A study of patients in Norway found that DLB patients had an SMR of 2.6 when compared to the general population [11]. However, this study excluded patients with an MMSE under 20, which could explain that the mortality found was slightly lower than that in our patient group. In two studies, survival was compared with a control group of non-DLB patients, showing increased hazard ratios of 8.3 [9] and 3.94 [12]. In the former, DLB patients only had a mean survival of 1.9 years and were significantly older than the non-demented controls, which might explain the considerably higher hazard ratio.

The majority of studies comparing survival times in AD and DLB have found increased mortality in DLB patients [3, 8, 11, 15, 43]. Even though this was not the focus of our study, reasonable comparison can be made with findings in the existing literature. Studies have reported an SMR of 1.50 for AD [11] and 1.49 for all-cause dementia (of which 37% were AD and 25% mixed AD-VaD) [3]. This is less than half of that seen in our study and others [11], similarly indicating increased mortality in patients with DLB/PDD. It is possible that factors predicting excess mortality are shared with other dementias, something which could be explored in future studies together with other clinical risk factor which could explain the discrepancy in mortality.

The major advantage of this study is that it is the first study to our knowledge utilizing relative survival regression models in this patient group. The study population is large and has one of the longest follow-up times. Further, we included all patients starting at the time when electronic medical records were implemented, not excluding patients on the basis of comorbidities or clinical features. This allowed for a heterogeneous group, which is representative of a memory clinic population, and should therefore make the results relevant to the practicing physician.

The date of diagnosis was verified in the electronic medical journals, and diagnoses were verified by a clinician according to consensus criteria which is another strength. Diagnostic accuracy is also suggested by the positive ^[123I]^FP-CIT SPECT scans, and that the post-mortem examinations confirmed the diagnosis in all available cases. Because of the unique Swedish social security numbers, we also know that the date of death is accurate and that national statistics used for the background data is trustworthy. Comorbidity burden in our population also seemed to be within the expected range based on current literature [15, 44].

We were limited by the clinical retrospective nature of this study, meaning that extended clinical data for all patients included was not available, such as cerebrospinal fluid analysis, neuroimaging, extended cognitive tests etc. This would be desirable for future analyses in order to identify other predictors of survival which could be useful in clinical practice.

Only patients who were referred to the memory clinic with a subsequent DLB or PDD diagnosis were included in this study, which could represent a selection bias. All suspected DLB patients should be referred to our specialist memory clinic according to regional guidelines, but patients with less characteristic features or those presenting later in the disease-course might be overlooked or remain undiagnosed [45]. Thus, the factors influencing the referral decision could affect the estimated mortality. Furthermore, patients with Parkinson’s disease are often managed by the neurology clinic, and only sometimes referred to the memory services at the onset of cognitive symptoms, explaining the smaller sample. To increase inclusion of PDD patients, future studies in our region could address this by also including patients from the neurology clinic and primary care services.

When using national life-tables for relative survival models, the disease under study will be included in the rates of expected mortality. This is only a problem in cases where the prevalence is high, and therefore not a concern for DLB/PDD in Sweden where the prevalence is approximately 0,4% in those over 65 years old [46, 47]. Even if this is less than the true prevalence, it will only lead to negligible bias [33] and an underestimation of true excess mortality. This is however an important aspect for future studies, particularly if investigating diagnoses that are highly prevalent in the population of interest, e.g. Alzheimer’s disease. Nevertheless, we believe that relative survival methodology has advantages for diagnoses other than cancer, and future studies could develop analyses by applying time-dependent effects and more complex modelling [48].

In view of the poor prognosis, our results have clinical implications as this also emphasizes the importance of a correct and timely diagnosis. This is relevant since DLB is still under-recognized in clinical practice [49]. Furthermore, the highest impact of the DLB disease on mortality has been found in patients who are younger, female and with suspected concomitant AD pathology. This information is important for practicing clinicians as well as policymakers, in order to be able to provide support and direct resources towards these patients in particular.

Other than worsened survival, as highlighted in this article, a diagnosis of DLB and PDD is associated with poor quality of life [50], high caregiver burden [51] and high healthcare costs [52]. To date, there is no disease-modifying therapy, and little evidence exists regarding non-pharmacological interventions [53]. Despite this, relatively few research efforts are focused toward this patient group [4].

In conclusion, mortality in patients diagnosed with dementia with Lewy bodies and Parkinson’s disease dementia is over three-times higher in patients during a ten-year follow-up, compared to persons in the general population unaffected by the disease. Excess mortality is found primarily in younger patients, females and carriers of *APOE* ε4. Further research is required regarding survival and possible interventions, including disease-modifying treatments, to improve care and prognosis for these patients.

## Supporting information

S1 File Approach to statistical analysis. (DOCX)

S2 File R code. (DOCX)
