# Supplementary material for: Relative survival in patients with dementia with Lewy bodies and Parkinson’s disease dementia
Source: PLoS One. 2018 Aug 10;13(8):e0202044. doi: 10.1371/journal.pone.0202044 (PMC6086429; doi:10.1371/journal.pone.0202044)
Supplement: S1 File — (DOCX) [file pone.0202044.s001.docx]

Supplement 1 – Statistical methods

The overall aim was to apply relative survival in a cohort of DLB and PDD patients, to get a better understanding of survival in this patient group compared to the general population.

**Data handling and computer programming**

All data was originally stored in an SPSS file. Survival analysis was performed in R.^1^ The script is provided in supplement 2.

**How to measure patient survival**

‘Survival curves’ is a frequently used term in the medical literature, referring to Kaplan Meier estimates plotted as survival probabilities against time. However, this is not the only and not always the most appropriate method to use, depending on the aim and population studied.

In most cases, clinicians are interested in the *disease-specific mortality*. However, this is problematic because of the limitations in obtaining this information, both because of a) poor reporting on death certificates and b) the difficulty in separating deaths unrelated from the disease of interest from indirect deaths. For example, should a fatal fall in a patient with Parkinson’s disease be recorded as unrelated to the disease of interest or an indirect result of the gait difficulties associated with the disease?

Because of this, many will instead record *all-cause mortality*, including all deaths occurring during the study. However, this means that deaths unrelated to the disease of interest will be measured. To then assess the true impact of a specific diagnosis is challenging, particularly in an aged population. Are the deaths occurring in the dementia population because of age and other comorbidities – or are they truly related to the dementia diagnosis?

Furthermore, age is a strong covariate in all-cause mortality analyses. This proves to be a limitation as the magnitude of the impact of age might differ between conditions. When adjusting for age, we will then mainly adjust for impact on one condition influencing mortality, rather than the one we are truly interested in.

An alternative method is analysis of *relative survival* or *excess mortality*.^2^ This estimates the mortality rate for the patients with the condition of interest by adjusting for mortality from all other causes, the so called *expected survival*. The expected survival rates are typically based on life-tables of the population of interest, organized by age, sex and calendar year. The relative survival rate is defined as a ratio of the observed survival rate in the cohort studied and the expected survival rate, based on a comparator population. The total mortality can therefore be defined as the expected mortality rate plus the excess mortality rate, irrespective if this is direct or indirect mortality related to the diagnosis of interest.

The standardized mortality ratio (SMR) is similar to excess mortality, describing the impact of the diagnosis by estimating the likelihood of death in patients with the diagnosis of interest compared to the general population.^3^ In comparison to relative survival methods, SMR does not provide any information on survival time or background mortality.^4^ However, it is a commonly used measure in clinical research to compare mortality in different populations, and is therefore used also in our study.

**Overall survival analysis**

Survival time was defined as the time from diagnosis to death or last follow-up (17 May 2017). Survival status was determined using the Swedish Population Registry at last follow-up. Those surviving at endpoint had been followed for a median of 72 months, with shortest follow-up 29 months and longest 206 months. For surviving males, median follow-up was 49 months and for surviving females 50 months. No patients were lost to follow-up. Figure A shows the baseline survival curve for the whole study population.


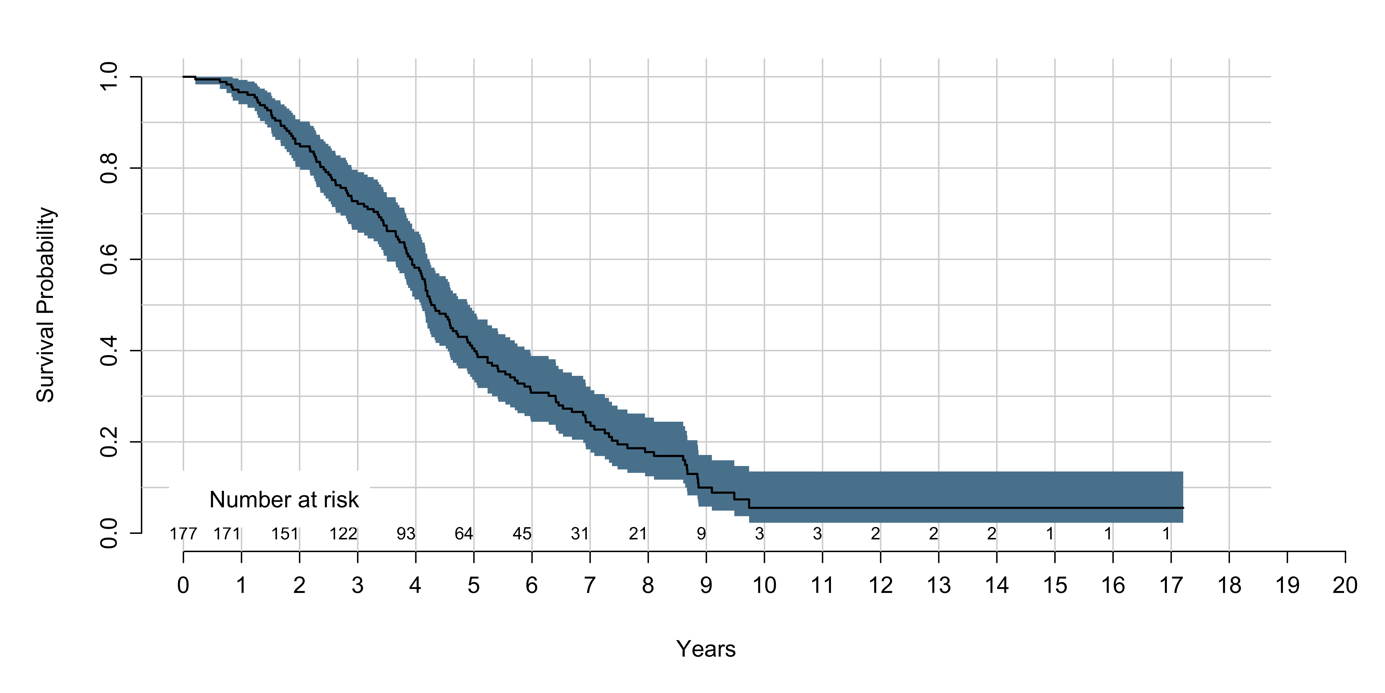


**Figure A. Baseline survival function for the full sample (n=177) with a 95% confidence interval and showing number at risk for the entire follow-up period.**

**Crude covariate analysis**

Bivariate Cox proportional hazards analysis was carried out for all the baseline variables to investigate the crude effect on survival (table A). Charlson co-morbidity index (CCI) was dichotomized into dementia only or additional co-morbidities. All other variables except were treated in their original form and scale. The data is presented with r coefficients, hazard ratios, 95% confidence intervals, standard errors, z-scores and p-values. Adjusting each variable for age- and sex was not significant. We also performed a multivariable Cox regression model, with no significant differences than in the univariate analysis (table B).

**Table A. Unadjusted bivariate Cox regressions for the separate baseline variables.**

| Variable | *β* | HR | 95% CI | | SE | z | p value |
| --- | --- | --- | --- | --- | --- | --- | --- |
| Age at diagnosis, years | 0.07 | 1.07 | 1.04 | 1.11 | 0.02 | 4.21 | <0.001 |
| Year at diagnosis | -0.01 | 0.99 | 0.94 | 1.04 | 0.03 | -0.36 | 0.72 |
| Presentation to diagnosis, months | 0.00 | 1.00 | 0.99 | 1.01 | 0.01 | -0.21 | 0.83 |
| Sex, 0=male, 1=female | -0.13 | 0.88 | 0.63 | 1.24 | 0.18 | -0.71 | 0.48 |
| Diagnosis, DLB = 0, PDD = 1 | -0.09 | 0.19 | 0.63 | 1.34 | 0.19 | -0.44 | 0.66 |
| Nursing home residency, 0=no, 1=yes | 0.55 | 1.74 | 1.00 | 3.03 | 0.28 | 1.95 | 0.05 |
| CCI, 0 = 0-1, 1= 2/more | 0.12 | 1.13 | 0.80 | 1.59 | 0.18 | 0.69 | 0.49 |
| MMSE score at diagnosis | -0.07 | 0.94 | 0.91 | 0.97 | 0.02 | -3.98 | <0.0001 |

**Abbreviations:** *β,* regression coefficient; SE, standard error; HR, hazard ratio; CI, confidence interval; DLB, dementia with Lewy bodies; PDD, Parkinson’s disease dementia; CCI, Charlson co-morbidity index; MMSE, mini-mental state examination.

**Table B. Multivariable Cox regression for baseline variables.**

| Variable | *β* | HR | 95% CI | | SE | z | p value |
| --- | --- | --- | --- | --- | --- | --- | --- |
| Age at diagnosis, years | 0.07 | 1.07 | 1.04 | 1.11 | 0.02 | 4.28 | <0.001 |
| Year at diagnosis | -0.01 | 0.99 | 0.94 | 1.04 | 0.03 | -0.45 | 0.66 |
| Presentation to diagnosis, months | 0.00 | 1.00 | 0.99 | 1.02 | 0.01 | 0.68 | 0.50 |
| Sex, 0=male, 1=female | -0.18 | 0.84 | 0.58 | 1.20 | 0.18 | -0.97 | 0.33 |
| Diagnosis, DLB = 0, PDD = 1 | -0.05 | 0.95 | 0.64 | 1.41 | 0.20 | -0.26 | 0.80 |
| Nursing home residency, 0=no, 1=yes | 0.20 | 1.22 | 0.67 | 2.20 | 0.30 | 0.66 | 0.51 |
| CCI score, 0 = 1, 1= 2/more | -0.01 | 0.99 | 0.68 | 1.43 | 0.19 | -0.06 | 0.95 |
| MMSE score at diagnosis | -0.07 | 0.93 | 0.90 | 0.96 | 0.02 | -4.30 | <0.001 |

**Abbreviations:** *β,* regression coefficient; SE, standard error; eHR, excess hazard ratio; CI, confidence interval; DLB, dementia with Lewy bodies; PDD, Parkinson’s disease dementia; CCI, Charlson co-morbidity index; MMSE, mini-mental state examination.

**Relative survival analysis**

The R package relsurv^5^ was used for all calculations of relative survival. The cumulative relative survival function is defined as the ratio between observed survival and expected survival in the relevant background population.

There are several methods to calculate the expected survival. We decided to use the method proposed by Hakulinen,^6^ as this has been recommended by others.^7^ Life-tables from the Swedish population were obtained from the Human Mortality Database (www.mortality.org), organized by sex, age and calendar year. Relative survival curves were calculated using the Pohar-Perme method.^8^

Cumulative survival rates are summarized in table C below. Compared to diagnoses with instant risk of dying e.g. acute MI,^9^ there is no increased risk of death in the early intervals after diagnosis.

**Table C. Cumulative overall, expected and relative survival for the whole group.**

| Interval, years | N start of interval | N deaths during interval | Cumulative observed survival | Cumulative expected | Relative cumulative survival |
| --- | --- | --- | --- | --- | --- |
| 0-1 | 177 | 6 | 0.966 | 0.957 | 1.010 |
| 1-2 | 171 | 20 | 0.853 | 0.914 | 0.933 |
| 2-3 | 151 | 23 | 0.722 | 0.871 | 0.828 |
| 3-4 | 122 | 23 | 0.582 | 0.827 | 0.703 |
| 4-5 | 93 | 28 | 0.405 | 0.783 | 0.517 |
| 5-6 | 64 | 15 | 0.308 | 0.742 | 0.415 |
| 6-7 | 45 | 9 | 0.243 | 0.702 | 0.346 |
| 7-8 | 31 | 8 | 0.178 | 0.665 | 0.267 |
| 8-9 | 21 | 8 | 0.100 | 0.632 | 0.158 |
| 9-10 | 9 | 3 | 0.056 | 0.609 | 0.091 |

The difference between overall, expected and relative survival is illustrated in figure 2 in the article. Relative survival is dependent on the expected mortality within the group studied. We have further illustrated this in figure B below, showing the difference between observed, expected and relative survival in two age groups with a cut-off at 75 years. As predicted, older patients will have a worse survival than younger patients, however as can be seen, some of this difference is attributed to increased background mortality and not due to worsened mortality due to the DLB/PDD diagnosis. There is consequently a larger discrepancy between observed and relative survival in the older age group.

**
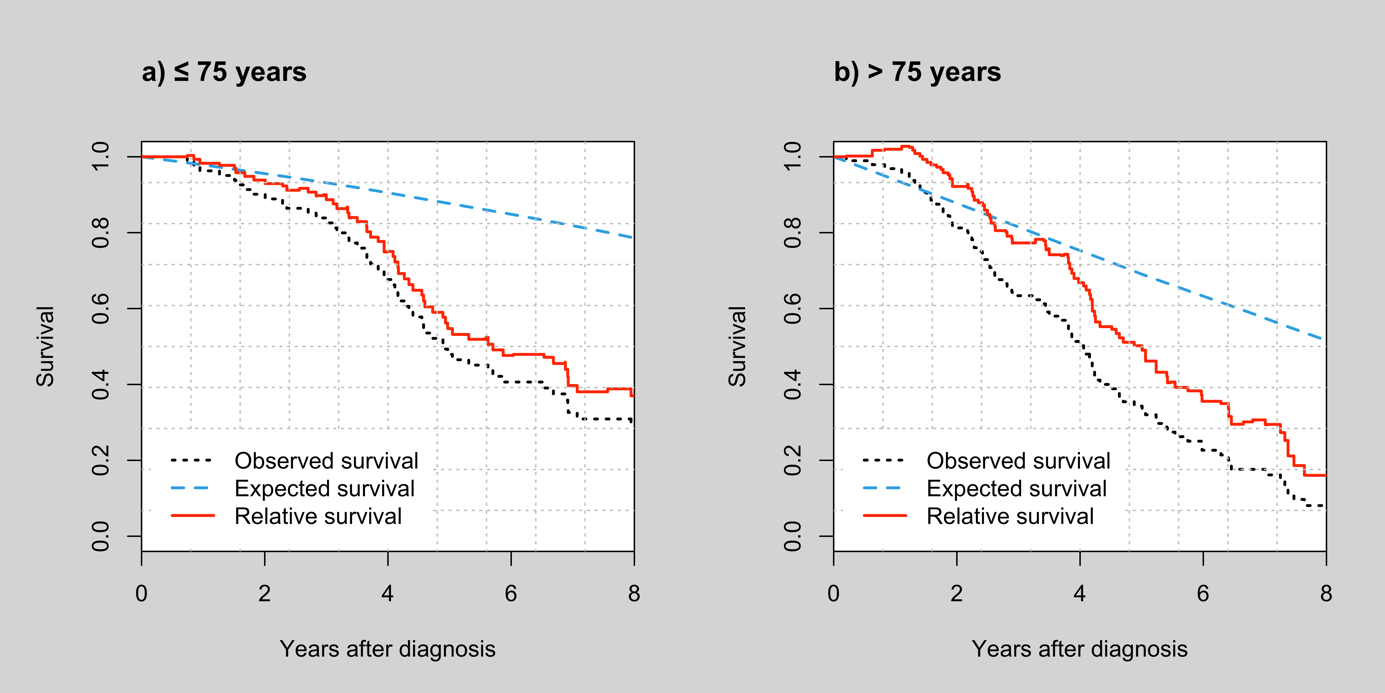
**

**Figure B. Observed, expected and relative survival in patients younger than 75 years and patients older than 75 years.**

**Relative regression models**

Estimation of excess hazard can be done in several different ways. The transformation method transforms all individual survival times to a new scale,^10^ taking in to account general population mortality, which we then can model similar to in Cox regression analysis. This yields excess hazard ratios (eHR) which specify the effect of the covariate on the excess mortality.

Separate analysis was carried out for all baseline variables, shown in table D. As in Cox regression, only age and MMSE (which is a proxy marker for cognitive level) are associated with excess hazard in this population.

**Table D. Excess hazard ratio for the separate baseline variables.**

|  | *β* | eHR | 95% CI | SE | z | p value |
| --- | --- | --- | --- | --- | --- | --- |
| Age diagnosis, years | -0.09 | 0.92 | 0.89-0.95 | 0.02 | -5.13 | <0.0001 |
| Year diagnosis | -0.01 | 0.99 | 0.95-1.04 | 0.02 | -0.25 | 0.81 |
| Presentation to diagnosis, months | 0.00 | 1.00 | 0.99-1.01 | 0.01 | 0.23 | 0.82 |
| Sex, 0=male, 1=female | 0.29 | 1.34 | 0.95-1.90 | 0.17 | 1.66 | 0.10 |
| Diagnosis, 0=DLB, 1=PDD | 0.07 | 1.08 | 0.74-1.58 | 0.19 | 0.40 | 0.69 |
| Nursing home residency, 0=no, 1=yes | 0.43 | 1.54 | 0.85-2.79 | 0.30 | 1.44 | 0.15 |
| CCI, 0 = 0-1, 1= 2/more | -0.09 | 0.92 | 0.65-1.30 | 0.18 | -0.50 | 0.62 |
| MMSE score at diagnosis | -0.07 | 0.93 | 0.90-0.96 | 0.02 | -4.30 | <0.0001 |

**Abbreviations:** *β,* regression coefficient; SE, standard error; eHR, excess hazard ratio; CI, confidence interval; DLB, dementia with Lewy bodies; PDD, Parkinson’s disease dementia; CCI, Charlson co-morbidity index; MMSE, mini-mental state examination.

To further assess the impact of covariates, multivariable relative survival regression modelling can be performed. In table E, we illustrate the different effects of modelling a variable using Cox regression and relative survival regression. Adjusting for age and year at diagnosis increases the eHR (from 1.34 to 1.46) in the relative model, whilst in the Cox regression it decreases (from 0.88 to 0.86). This is because females have an increased expected survival compared to males and resulting in relatively more lives lost to the disease of interest.

**Table E. Comparison of Cox multivariable analysis and relative multivariable analysis.**

| A) Cox multivariable regression analysis | | | | | | |
| --- | --- | --- | --- | --- | --- | --- |
|  | ***β*** | **HR** | **95% CI** | **SE** | **z** | **p value** |
| Age at diagnosis, years | 0.07 | 1.07 | 1.04-1.11 | 0.07 | 4.22 | <0.0001 |
| Sex, male = 0, female = 1 | -0.15 | 0.86 | 0.61-1.22 | 0.18 | -0.85 | 0.397 |
| Year at diagnosis | -0.01 | 0.10 | 0.94-1.04 | 0.03 | -0.41 | 0.67 |
| B) Relative survival multivariable regression analysis | | | | | | |
|  | ***β*** | **eHR** | **95% CI** | **SE** | **z** | **p value** |
| Age at diagnosis, years | -0.09 | 0.91 | 0.89-0.95 | 0.02 | -5.29 | <0.0001 |
| Sex, male = 0, female = 1 | 0.38 | 1.46 | 1.02-2.08 | 0.18 | 2.09 | 0.04 |
| Year at diagnosis | 0.022 | 1.02 | 0.97-1.07 | 0.03 | 0.86 | 0.39 |

**Abbreviations:** *β,* regression coefficient; SE, standard error; HR, hazard ratio; CI, confidence interval.

We constructed a multivariable relative survival model of all the baseline variables (table 2 in article), excluding *APOE* ɛ4 which was analysed separately.

**Comparison between DLB and PDD patients**

No differences in survival was found between DLB and PDD patients, illustrated by Kaplan-Meier curve below (figure C). This was true also after adjusting survival for sex and age and diagnosis, as seen in table F.

**
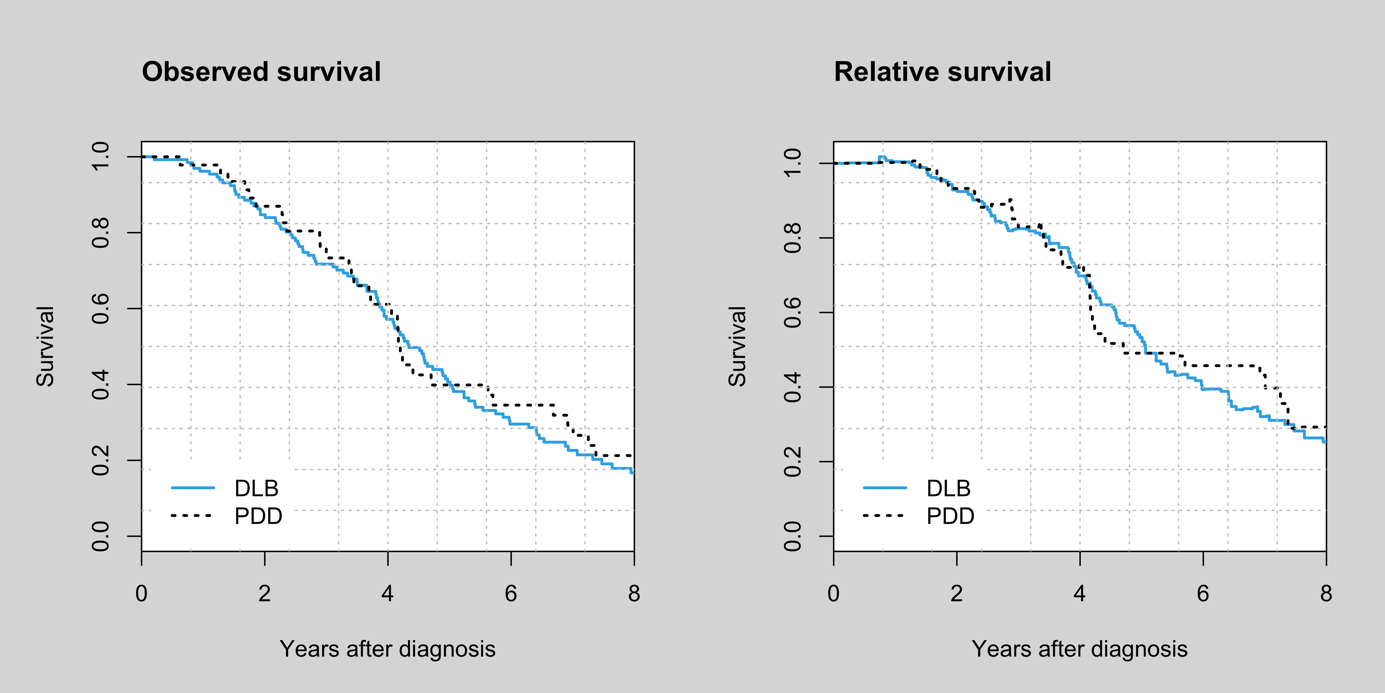
**

**Figure C. Observed survival in DLB (n=131) and PDD (n=46) patients.**

**Table F. Age- and sex-adjusted Cox and relative regression analysis comparing diagnoses***.*

| A) Cox multivariable regression analysis | | | | | | |
| --- | --- | --- | --- | --- | --- | --- |
|  | ***β*** | **HR** | **95% CI** | **SE** | **z** | **p value** |
| Age at diagnosis, years | 0.07 | 1.07 | 1.04-1.11 | 0.02 | 4.19 | <0.0001 |
| Sex, 0=male, 1=female | -0.14 | 0.87 | 0.61-1.22 | 0.18 | -0.82 | 0.42 |
| Diagnosis, 0=DLB, 1=PDD | -0.02 | 0.98 | 0.67-1.44 | 0.20 | -0.10 | 0.92 |
| B) Relative survival multivariable regression analysis | | | | | | |
|  | ***β*** | **eHR** | **95% CI** | **SE** | **z** | **p value** |
| Age at diagnosis, years | -0.09 | 0.92 | 0.89-0.94 | 0.02 | -5.25 | <0.0001 |
| Sex, 0=male, 1=female | 0.36 | 1.43 | 1.01-2.04 | 0.18 | 2.02 | 0.04 |
| Diagnosis, 0=DLB, 1=PDD | -0.01 | 0.99 | 0.68-1.46 | 0.20 | -0.03 | 0.98 |

**Abbreviations:** *β,* regression coefficient; SE, standard error; HR, hazard ratio; CI, confidence interval.

**Goodness of fit**

Model fit was tested using the recommended method in relative survival modelling, based on Schoenfeld-type residuals.^11^ The proportionality assumption was not violated for the model shown in table 2 in the article (p=0.241). This is further illustrated in figure D below.


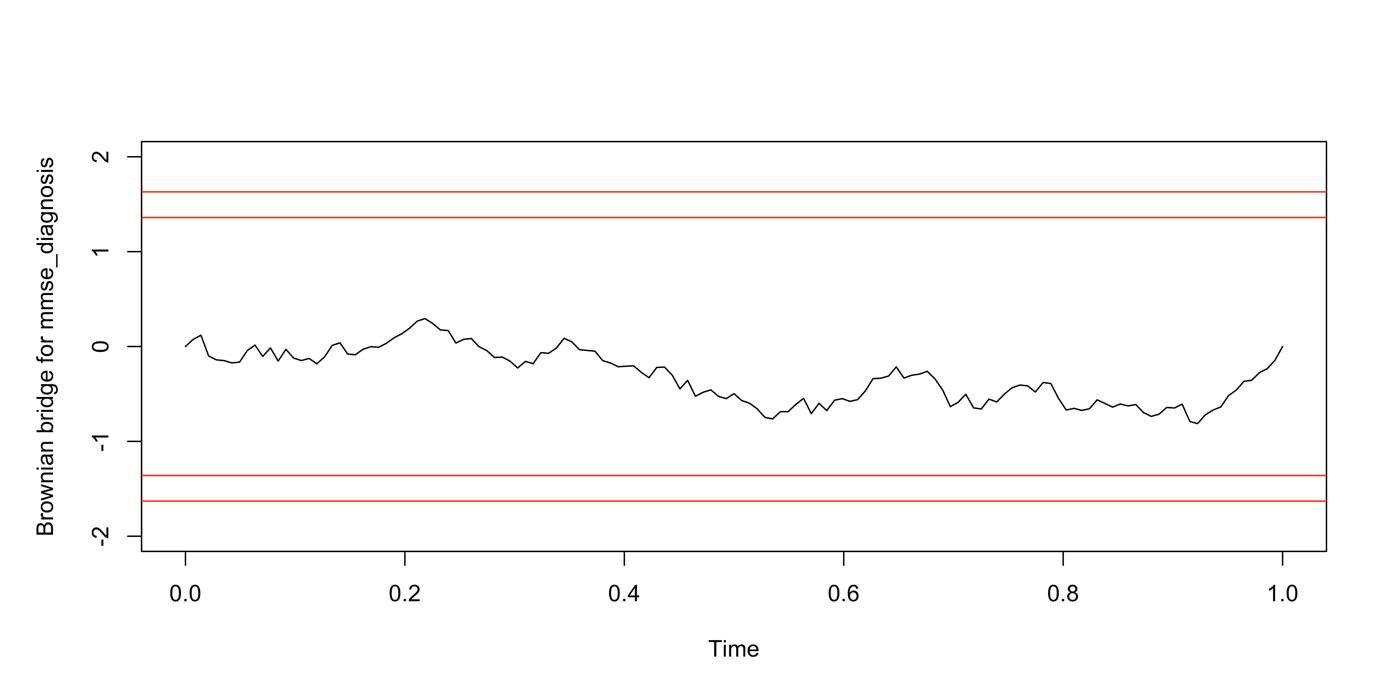


**Figure D. Brownian bridge processes for the global model corresponding to the fit of model in table 2 in the article.** Horizontal lines represent 95 and 99 percent values for the distribution.

**Missing data**

Missing values are common in clinical studies. It is important to investigate the quantities of missing data and if they are missing at random or if there are any patterns, as this can influence the results.

In our population, the dataset was complete for all the baseline variables except *APOE* analysis where n=141. Patients with missing data for *APOE* were on average older, diagnosed later in time and more often PDD patients. The explanation comes from the clinical routines in our clinic, where patients will only be routinely analysed for *APOE* when being referred for cerebrospinal fluid examination, something which is sometimes omitted in elderly patients. Furthermore, in patients with Parkinson’s disease and a suspicion of PDD, a lumbar puncture is less frequently carried out than in patients with DLB where the diagnosis might initially be more uncertain.

**Table G. Missing data analysis.**

| Variable | Present | Missing | p-value |
| --- | --- | --- | --- |
| Age at diagnosis, years | 74.8 | 79.0 | <0.001 |
| Year of diagnosis | 2007 | 2009 | 0.009 |
| Presentation to diagnosis, months | 38.8 | 21.1 | 0.052 |
| MMSE score at diagnosis | 21.9 | 22.8 | 0.260 |
| Charlson co-morbidity index | 1.4 | 1.7 | 0.161 |
| Gender (male %) | 63 | 69 | 0.479 |
| Diagnosis (DLB %) | 78 | 58 | 0.016 |

**Abbreviations:** *APOE*, apolipoprotein E; DLB, dementia with Lewy bodies.

There are a number of ways of handling missing data including simple and complex imputation techniques. However, *APOE* is measured only once in each individual, and the variables associated with outcome can all be included as covariates (diagnosis, age, year), and therefore analyses will not be biased.^12^ For this reason, we have analysed patients with *APOE* status separately, i.e. complete-case analysis only. No differences were found between in the baseline variables between carriers and non-carriers (table H below).

**Table H. Comparison of baseline variables in APOE ɛ4 carriers and non-carriers.**

| Variable | Carriers | Non-carriers | p-value |
| --- | --- | --- | --- |
| Age at diagnosis, years (±SD) | 73.9 (5.6) | 75.7 (5.6) | 0.062 |
| Presentation to diagnosis, months (IQR) | 4 (0-7) | 2 (0-7) | 0.450 |
| Year of diagnosis (SD) | 2007 (3) | 2007 (3) | 0.846 |
| Male sex (%) | 57 | 67 | 0.134 |
| Diagnosis DLB:PDD (%) | 82:18 | 74:26 | 0.266 |
| CCI points (IQR) | 1 (1-2) | 1 (1-2) | 0.997 |
| MMSE at diagnosis, points | 21.5 | 22.3 | 0.314 |

**Abbreviations:** DLB, dementia with Lewy bodies; PDD, Parkinson’s disease dementia; CCI, Charlson co-morbidity index; *APOE*, apolipoprotein E; MMSE, mini-mental state examination.

References

1. R: A language and environment for statistical computing. [program]. Vienna, Austria: Foundation for Statistical Computing, 2016.

2. Ederer F, Axtell LM, Cutler SJ. The relative survival rate: a statistical methodology. *Natl Cancer Inst Monogr* 1961;6:101-21.

3. Breslow NE, Day NE. Statistical methods in cancer research. Volume II--The design and analysis of cohort studies. *IARC Sci Publ* 1987(82):1-406.

4. Smoll NR, Gautschi OP, Radovanovic I, et al. Incidence and relative survival of chordomas: the standardized mortality ratio and the impact of chordomas on a population. *Cancer* 2013;119(11):2029-37. doi: 10.1002/cncr.28032

5. relsurv: Relative Survival. R package version 2.0-9. [program], 2016.

6. Hakulinen T. Cancer survival corrected for heterogeneity in patient withdrawal. *Biometrics* 1982;38(4):933-42.

7. Pohar M, Stare J. Making relative survival analysis relatively easy. *Comput Biol Med* 2007;37(12):1741-9. doi: 10.1016/j.compbiomed.2007.04.010

8. Pohar M, Stare J. Relative survival analysis in R. *Comput Methods Programs Biomed* 2006;81(3):272-8. doi: 10.1016/j.cmpb.2006.01.004

9. Nelson CP, Lambert PC, Squire IB, et al. Relative survival: what can cardiovascular disease learn from cancer? *Eur Heart J* 2008;29(7):941-7. doi: 10.1093/eurheartj/ehn079

10. Stare J, Henderson R, Pohar M. An individual measure of relative survival. *Applied Statistics* 2005;54(1):115-26. doi: 10.1111/j.1467-9876.2005.00473.x

11. Stare J, Pohar M, Henderson R. Goodness of fit of relative survival models. *Stat Med* 2005;24(24):3911-25. doi: 10.1002/sim.2414

12. Sterne JA, White IR, Carlin JB, et al. Multiple imputation for missing data in epidemiological and clinical research: potential and pitfalls. *BMJ* 2009;338:b2393. doi: 10.1136/bmj.b2393
