# Supplementary material for: Relative survival in patients with dementia with Lewy bodies and Parkinson’s disease dementia
Source: PLoS One. 2018 Aug 10;13(8):e0202044. doi: 10.1371/journal.pone.0202044 (PMC6086429; doi:10.1371/journal.pone.0202044)
Supplement: S2 File — (DOCX) [file pone.0202044.s002.docx]

Supplement 2 – R code survival analysis

# Load packages

library(haven)

library(Hmisc)

library(rms)

library(gmodels)

library(survival)

library(relsurv)

# Data import from SPSS

survdata <- read_sav() #file path in brackets to load data

survdataapoe <- read_sav() #file path to subgroup with apoe analysis

# Load population table

swepop <- transrate.hmd (male = file path.txt, female = file path.txt)

attributes(swepop)$dimid

summary(swepop)

# OVERALL SURVIVAL ANALYSIS

# Determining follow-up and censoring

S <- Surv(time_months, event)

cens.time <- ifelse(event == 0, time_months, NA)

summary(cens.time)

# Survival depending on gender

maletime <- ifelse(sex==1, time_months,NA)

summary(maletime)

femaletime <- ifelse(sex==2, time_months,NA)

summary(femaletime)

# Baseline survival plot, figure 1 supplement 1

overallsurv <- Surv(time_days/365.24, event)

survplot(npsurv(overallsurv~1))

# Univariate analysis for predictors of survival, table 2 article

summary(coxph(S~age_diagnosis))

summary(coxph(S~year_diagnosis))

summary(coxph(S~time_delay))

summary(coxph(S~sex))

summary(coxph(S~diagnosis))

summary(coxph(S~nursinghome))

summary(coxph(S~cci_dic))

summary(coxph(S~mmse_diagnosis))

summary(coxph(S~apoe4))

# Multivariate analysis for predictors of survival, table 1 supplement 1

coxmodel <-coxph(S~age_diagnosis+year_diagnosis+time_delay+sex

+diagnosis+nursinghome+cci_dic+mmse_diagnosis, data=survdata)

summary(coxmodel)

# RELATIVE SURVIVAL ANALYSIS

#Survival rates, table 2 supplement 1

obs <- survfit(Surv(time_days,event)~1,data=survdata) #observed survival

summary(obs, times=0:10*365.241)

exp <- survexp(time_days~ 1, rmap= list(year=year, age=age_diagnosis_days, sex=sex),

method='hakulinen', data=survdata, ratetable=swepop) # expected survival

summary(exp, times=0:15*365.241)

rel<-rs.surv(Surv(time_days,event)~ ratetable(age=age_diagnosis_days,

sex=sex, year=year), data=survdata, ratetable=swepop) #relative survival

summary(rel, times=0:15*365.241)

# Figure 2 article

tiff(filename="FIG2.TIF", res=1000, compression = "lzw", height=70, width=140, units="mm", pointsize=7)

par(mar=c(5,5,2,2)+0.1, bg='lightgray')

plot(obs, xscale=365.241, xlim=c(0,10), xlab="Years after diagnosis", ylab="Survival")

x<-par("usr")

rect(x[1],x[3],x[2],x[4],col="white")

lines(obs, main=NULL, sub=NULL, mark.time=F, bty="n", lwd=1, lty=3, conf.int=F)

lines(exp, col="#2E9FDF", lwd=1, lty=2) # Add the expected survival line

lines(rel, col=2, conf.int = F, lwd=1) # Add the relative survival line

grid(nx=10, col="grey", lwd=0.5)

lnames <- c('Observed survival', 'Expected survival', 'Relative survival')

legend('topright', lnames, col = c(1,"#2E9FDF", 2), lty = c(3,2,1), lwd=1, inset=0.02, bty="o", bg="white", box.col="white")

dev.off()

#Individual eHR for each baseline variable, table 3 supplement 1

summary(rstrans(Surv(time_days,event)~age_diagnosis+ratetable(age=

age_diagnosis*365.24,sex=sex,year=year),data=survdata,ratetable=swepop))

summary(rstrans(Surv(time_days,event)~time_delay+ratetable(age=

age_diagnosis*365.24,sex=sex,year=year),data=survdata,ratetable=swepop))

summary(rstrans(Surv(time_days,event)~sex+ratetable(age=

age_diagnosis*365.24,sex=sex,year=year),data=survdata,ratetable=swepop))

summary(rstrans(Surv(time_days,event)~year_diagnosis+ratetable(age=

age_diagnosis*365.24,sex=sex,year=year),data=survdata,ratetable=swepop))

summary(rstrans(Surv(time_days,event)~diagnosis+ratetable(age=

age_diagnosis*365.24,sex=sex,year=year),data=survdata,ratetable=swepop))

summary(rstrans(Surv(time_days,event)~nursinghome+ratetable(age=

age_diagnosis*365.24,sex=sex,year=year),data=survdata,ratetable=swepop))

summary(rstrans(Surv(time_days,event)~cci_dic+ratetable(age=

age_diagnosis*365.24,sex=sex,year=year),data=survdata,ratetable=swepop))

summary(rstrans(Surv(time_days,event)~mmse_diagnosis+ratetable(age=

age_diagnosis*365.24,sex=sex,year=year),data=survdata,ratetable=swepop))

# Comparison Cox vs relative survival multivariable models, table 4 supplement 1

coxmodel <-coxph(S~age_diagnosis+year_diagnosis+sex, data=survdata)

summary(coxmodel)

relmodel <- rstrans(Surv(time_days,event)~age_diagnosis+year_diagnosis

+sex+ratetable(age=age_diagnosis_days,

sex=sex, year=year), data=survdata, ratetable=swepop)

summary(relmodel)

# Multivariable relative survival model, table 3 article

relmodel2 <- rstrans(Surv(time_days,event)~age_diagnosis+year_diagnosis+time_delay+sex

+diagnosis+nursinghome+cci_dic+mmse_diagnosis+ratetable(age=age_diagnosis_days,

sex=sex, year=year), data=survdata, ratetable=swepop)

summary(relmodel2)

# GOODNESS OF FIT

rsbr <- rs.br(relmodel2)

tiff(filename="rsbr", res=600, compression = "lzw", height=5, width=10, units="in")

par(mar=c(5,5,5,2)+0.1, bg='white',mfrow=c(1,1))

plot(rsbr)

dev.off()

# MISSING VALUES ANALYSIS

t.test(age_diagnosis~missingapoe)

t.test(year_diagnosis~missingapoe)

t.test(time_delay~missingapoe)

t.test(mmse_diagnosis~missingapoe)

CrossTable(sex, missingapoe, chisq=T)

CrossTable(diagnosis, missingapoe, chisq=T)

CrossTable(cci_dic, missingapoe, chisq=T)

# SUBGROUP ANALYSIS

# Overall survival

summary(coxph(S~apoe4)) #unadjusted

apoeint <-coxph(Surv(time_months,event)~factor(diagnosis*apoe4)

+age_diagnosis+sex, data =survdataapoe) #coxmodel with interaction

summary(apoeint)

#Excess hazard ratio

rsapoe4 <- rstrans(Surv(time_days,event)~apoe4

+ratetable(age=age_diagnosis*365.24,sex=sex,year=year),

data=survdata,ratetable=swepop) #unadjusted

summary(rsapoe4)

intrs <- rstrans(Surv(time_days,event)~factor(apoe4*diagnosis)

+sex+age_diagnosis+ratetable(age=age_diagnosis*365.24

,sex=sex,year=year),data=survdataapoe,ratetable=swepop) #interaction

summary(intrs2)

# STANDARDIZED MORTALITY RATIO

risk=-log(survexp(time_days~ 1, data=survdata,rmap=list(year=year,

             age=age_diagnosis_days, sex=sex),

             method='individual.s', ratetable=swepop)) #individual expected

E=sum(risk) #expected deaths

O=sum(event) #observed deaths

O/E #smr

O/E*(1-1/9/O-qnorm(1-0.05/2)/3/sqrt(O))^3 #CI lo

(O+1)/E*(1-1/9/(O+1)+qnorm(1-0.05/2)/3/sqrt(O+1))^3 #CI up

1-pchisq(chisq,1) #pvalue
